# Trends in heart failure prevalence in post-disaster Fukushima residents 2015–2021

**DOI:** 10.1038/s41598-026-36032-0

**Published:** 2026-01-14

**Authors:** Enbo Ma, Tetsuya Ohira, Maiko Fukasawa, Atsushi Shirafuji, Hiromasa Ohira, Michio Shimabukuro

**Affiliations:** 1 Health Promotion Center, Fukushima Medical University, Fukushima City, Fukushima 960-1295 Japan; 2 Department of Epidemiology, Fukushima Medical University School of Medicine, Fukushima City, Fukushima 960-1295 Japan; 3 Radiation Medical Science Centre for the Fukushima Health Management Survey, Fukushima Medical University, Fukushima City, Fukushima 960-1295 Japan; 4 Department of Computer Science and Engineering, The University of Aizu, Aizuwakamatsu City, Fukushima 965-8580 Japan; 5 Department of Gastroenterology, Fukushima Medical University School of Medicine, Fukushima City, Fukushima 960-1295 Japan; 6 Department of Diabetes, Endocrinology and Metabolism, Fukushima Medical University School of Medicine, Fukushima City, Fukushima 960-1295 Japan

**Keywords:** Heart failure, Prevalence, Disaster, Fukushima, Diseases, Health care, Medical research, Risk factors

## Abstract

This study aimed to investigate the prevalence of heart failure (HF) among adults aged ≥ 40 years using health checkup and medical claim data in Fukushima from 2015 to 2021. Joinpoint regression and age-period-cohort analyses were conducted to estimate temporal trends. Age-standardized prevalence and hospital admission rates for HF were 37.0 and 7.4/1000 and 25.9 and 5.3/1000 for men and women, respectively. The prevalence was significantly higher in the coastal area and evacuation zone designated after the 2011 disaster compared to the prefecture overall. In men, the prevalence increased continuously, with an average annual percentage change (AAPC) ranging from 0.72% (evacuation zone) to 1.15% (mountainous area) (*P* < 0.05). In total residents, the AAPC was significant only in the mountainous areas (0.78%, *P* = 0.021). Age-period-cohort analysis showed a net drift of 2.50% (95% CI 1.88–3.13%) in men and 0.76% (95% CI − 0.17–1.70%) in women. Cohort rate ratios increased significantly in men born between 1925 and 1975, while in women, they decreased for those born between 1925 and 1960 but increased for those born between 1960 and 1970. The prevalence of HF varied across post-disaster areas of Fukushima. Given that pathological changes and modifiable risk factors for HF accumulate gradually, continuous monitoring among middle-aged adults is essential to enable timely prevention and targeted intervention.

**Supplementary Information:**

The online version contains supplementary material available at 10.1038/s41598-026-36032-0.

## Introduction

Heart failure (HF) is a major clinical and public health concern^1^, especially in Japan due to its ageing population^2,3^. HF occurs in cycles of exacerbation, common multimorbidities, and remission, causing frequent hospitalizations and increased financial burden^4–6^. The number of Japanese outpatients with left ventricular dysfunction is estimated to reach 1.3 million by 2030^7^.

There is a scarcity of population-based reports on HF in Japan^6,8,9^. Nationwide studies in Japan are mainly hospital-based studies from registries, such as the Japanese Registry of All Cardiac and Vascular Diseases (JROAD)^10^, Japanese Heart Failure Syndrome With Preserved Ejection Fraction Registry^11^, Japanese Society of Cardiovascular Physical Therapy Multicenter Registry of Older Frail patients with HF^12^, Chronic Heart Failure Analysis and Registry in the Tohoku District-1 (CHART-1) and -2 Study (CHART-2)^3,13^, and West Tokyo Heart Failure registry^14^.

The National Database of Health Insurance Claims and Specific Health Checkups of Japan (NDB) has been utilized to investigate health issues, facilitating comparisons among prefectures and sub-areas^15,16^. The Fukushima Health Database (FDB) was created in 2017, serving the same purpose as the NDB and supporting local health authorities in implementing evidence-based health promotion strategies. Health insurance is universal in Japan. The FDB includes residents across all 59 municipalities in the prefecture, regardless of insurance type. A validation study comparing the prevalence of metabolic risk factors in the FDB with those in the NDB demonstrated highly similar results^17^. Studies based on the NDB^16^, the Fukushima Health Management Survey^18^, and the FDB^17^ have consistently reported increases in metabolic cardiovascular risk factors, including overweight/obesity, hypertension, dyslipidemia, and elevated fasting plasma glucose, among residents of Fukushima in both evacuation and non-evacuation areas, following the Great East Japan Earthquake. However, a specific relevant health indicator, such as the prevalence of HF, has not been explored or reported.

The FDB participants were mainly those with national health insurance (NHI) (covering the unemployed, self-employed, and those in the agriculture, forestry, or fishery industry aged < 75 years), employee health insurance (EHI) (covering mainly employees of small- and medium-sized companies and dependents aged < 75 years), or latter-stage elderly health insurance (LEHI, aged ≥ 75 years). The NHI and EHI cover approximately 84.4% of Fukushima residents aged 40–74 years in 2021^19^. The proportion of participants who underwent the annual health checkup in Fukushima in 2021 was 56.3%, the same as the national average^20^. In addition, a recent report indicated that 76.4% of the prefectural population aged 40–74 years in 2022 were enrolled in NHI, EHI, and LEHI^21^. This study used data from a health insurance database representative of the regional population, focusing on the major insurance schemes, to examine the prevalence and trends of HF.

## Methods

### Study participants

Age- and sex-specific data for HF in Fukushima were retrieved from the FDB; these data were entered during participants’ health checkup sessions. The data of participants aged ≥ 40 years between 2015 and 2021 were included in the primary analysis. In total, 2,658,637 FDB participants who had attended at least one health checkup during 2015 and 2021 were included in this study; participants with NHI, EHI, and LEHI accounted for 36.2%, 46.1%, and 17.7%, respectively.

### Heart failure cases

The medical claim data were used to identify HF cases by requiring the presence of both diagnosis codes from the 10th International Classification of Diseases (ICD-10) for HF (I50.0–I50.9) and prescription records for HF-related medications, classified according to the Japanese Drug Efficacy Classification System (211, cardiotonic; 212, irregular pulse controller; 213, diuretic; 214, hypotensive drug; 217, vasodilator; and 333, anticoagulation)^22^. Because the claims data may include repeated outpatient visits or hospitalizations for the same individual, we counted only the first recorded HF case for each individual within a given fiscal year when calculating prevalence.

As medical claims data for 2012–2021 were available for NHI participants of all ages and LEHI participants in the FDB, an additional analysis by insurance type was conducted for data collected before 2015.

### Study subareas

Fukushima Prefecture is divided into three regions^16^: the mountainous area located on the west side, the central area with prominent cities, and the coastal area along the eastern coastline, including the nuclear power plant and the majority of evacuation zones. The evacuation zones were designed because of nuclear power plant accidents in March 2011; these include Tamura City, Minamisoma City, Kawamata Town, Hirono Town, Naraha Town, Tomioka Town, Kawauchi Village, Okuma Town, Futaba Town, Namie Town, Katsurao Town, and Iitate Village^16,19^. To ensure non-overlapping classification, regions were grouped into mountainous, central, coastal, and evacuation areas, with residents of the evacuation zones excluded from the other areas^16^.

The study was conducted in accordance with the ethical principles outlined in the Declaration of Helsinki and was approved by the Fukushima Medical University Ethical Review Committee (Generic 2021 - 169). The study was conducted using secondary administrative data accumulated in the database, with personal information anonymized. Informed consent was waived by the Fukushima Medical University Ethical Review Committee.

### Statistical analysis

We measured the annual prevalence rate of HF (per 1000 individuals), defined as the proportion of participants with a recorded diagnosis of HF, counting each individual only once if multiple inpatient or outpatient records were present. We also calculated the annual hospitalization rate of HF (per 1000 individuals), defined as the number of hospital admissions for HF among all health checkup participants.

#### Standardized prevalence rate

Age-standardized prevalence rates for each year were calculated using the country’s 2015 census as a reference for those aged 40–85 + years. The overall difference in standardized prevalence rates among areas in Fukushima was examined using generalized linear regression models adjusted for sex and study years.

#### Joinpoint regression analysis

The temporal trends of age-standardized HF prevalence rates were estimated using Joinpoint Regression analysis (version 5.2.0), developed by the US National Cancer Institute (NCI) for their surveillance research program^23^. Using Joinpoint regression models, annual percentage change (APC), average annual percentage change (AAPC), and 95% confidence intervals (CIs) were calculated. Monte Carlo permutation tests were used to test the significance of increasing (APC > 0) and decreasing trends (APC < 0).

#### Age-period-cohort analysis

Age-period-cohort analysis was used to identify the effects of age, period, and cohort on the trends in HF prevalence. We classified the data into one-year interval age categories from 40 to 89 years and one-year interval period categories from 2015 to 2021. A web tool implemented in the R code developed by the US NCI was used for the analysis^24^. The net drift (the estimated APC in expected age-adjusted rates) and local drift (the estimated APC in age-specific rates over time) were estimated, and longitudinal age curves (showing the expected age-specific rates adjusted for period effects in the reference cohort) were obtained. In the age-period-cohort analysis, the period rate ratio (RR) was used to evaluate the impact of the period, representing how age-specific rates changed in different periods compared with the reference period (p0). Cohort RR assessed the impact of the birth cohort, illustrating the age-specific prevalence rates for different cohorts compared with a reference cohort (c0). We used the middle period (p0 = 2018) and cohort (c0 = 1955) as references. In addition, period and cohort deviations (departing from overall linearity), observed rates being higher or lower than the expected rate based on age, period, and cohort effects, were also calculated.

Except for Joinpoint regression and age-period-cohort analyses, SAS 9.4 version (SAS Institute Inc., Cary, NC, USA) was used for data preparation and analysis. All statistical tests were two-sided, and a *P* value < 0.05 was considered significant.

## Results

A total of 1,287,130 men (48.4%) and 1,371,507 women (51.6%) aged ≥ 40 years attended at least one annual health checkup between 2015 and 2021. Table 1 shows the annual characteristics of the FDB participants. Women were older, had a lower BMI, and had fewer cardiovascular and renal diseases than men. Both men and women had a low prevalence of cerebrovascular disease over the years. The number of women treated for hypertension and type 2 diabetes mellitus was less than the number of men treated for the same. The number of women with hyperlipidemia treatment was higher. The crude HF prevalence in men (3.5–4.1%) was higher than that in women (2.5–2.7%).

**Table 1:** Characteristics of health checkup participants in the Fukushima Health Database, 2015–2021.

	2015		2016		2017		2018		2019		2020		2021	
Men^a^, No.	170,257		174,538		184,390		190,436		192,214		180,097		195,198	
Mountainous, No. (%)	28,038	(16.5)	28,014	(16.1)	28,952	(15.7)	29,583	(15.5)	29,867	(15.5)	27,356	(15.2)	29,691	(15.2)
Central, No. (%)	91,814	(53.9)	94,887	(54.4)	99,567	(54.0)	103,013	(54.1)	105,376	(54.8)	98,409	(54.6)	107,501	(55.1)
Coastal, No. (%)	28,502	(16.7)	28,681	(16.4)	30,937	(16.8)	31,616	(16.6)	30,848	(16.0)	29,332	(16.3)	31,282	(16.0)
Evacuation, No. (%)	15,795	(9.3)	16,056	(9.2)	16,815	(9.1)	17,252	(9.1)	16,913	(8.8)	15,736	(8.7)	16,997	(8.7)
Age, mean (SD), year	62.1	(12.3)	62.1	(12.4)	62.1	(12.5)	62.2	(12.5)	62.2	(12.5)	61.9	(12.5)	62.2	(12.6)
Body mass index, mean (SD), kg/m^2^	23.9	(3.4)	24.0	(3.4)	24.1	(3.5)	24.1	(3.5)	24.2	(3.5)	24.3	(3.6)	24.3	(3.6)
Medical history														
Cerebrovascular disease, No. (%)	12,644	(7.4)	6785	(3.9)	7121	(3.9)	7189	(3.8)	7316	(3.8)	5374	(3.0)	5675	(2.9)
Heart disease, No. (%)	8443	(5.0)	11,479	(6.6)	12,218	(6.6)	13,112	(6.9)	13,982	(7.3)	10,155	(5.6)	11,164	(5.7)
Renal disease, No. (%)	2868	(1.7)	595	(0.3)	725	(0.4)	1326	(0.7)	1708	(0.9)	1258	(0.7)	1466	(0.8)
Hypertension therapy, No. (%)	60,955	(37.6)	65,142	(37.3)	69,133	(37.5)	72,307	(38.0)	73,962	(38.5)	58,811	(36.2)	65,110	(37.0)
Diabetes therapy, No. (%)	18,571	(11.5)	19,581	(11.2)	21,551	(11.7)	23,242	(12.2)	23,883	(12.4)	20,243	(12.5)	22,400	(12.7)
Hyperlipidemia therapy, No. (%)	26,146	(16.1)	27,985	(16.0)	30,635	(16.6)	34,471	(18.1)	36,423	(19.0)	30,796	(19.0)	34,912	(19.8)
Current heart failure, No. (%)	6041	(3.6)	6097	(3.5)	6567	(3.6)	6896	(3.6)	7287	(3.8)	6897	(3.8)	7902	(4.1)
Women^a^, No.	184,107		189,102		200,124		204,116		205,552		185,187		203,319	
Mountainous, No. (%)	31,343	(17.0)	31,676	(16.8)	32,583	(16.3)	33,351	(16.3)	33,380	(16.2)	29,862	(16.1)	32,040	(15.8)
Central, No. (%)	99,403	(54.0)	102,922	(54.4)	108,592	(54.3)	111,293	(54.5)	113,642	(55.3)	102,301	(55.2)	113,664	(55.9)
Coastal, No. (%)	31,196	(16.9)	31,174	(16.5)	33,769	(16.9)	33,810	(16.6)	32,937	(16.0)	29,850	(16.1)	32,474	(16.0)
Evacuation, No. (%)	17,517	(9.5)	18,224	(9.6)	18,790	(9.4)	19,020	(9.3)	18,615	(9.1)	16,545	(8.9)	17,936	(8.8)
Age, mean (SD), year	63.9	(12.2)	63.9	(12.3)	63.7	(12.4)	63.8	(12.5)	63.8	(12.5)	63.4	(12.6)	63.6	(12.7)
Body mass index, mean (SD), kg/m^2^	22.9	(3.7)	23.0	(3.8)	23.0	(3.8)	23.1	(3.8)	23.1	(3.9)	23.1	(3.9)	23.1	(4.0)
Medical history														
Cerebrovascular disease, No. (%)	10,639	(5.8)	4382	(2.3)	4525	(2.3)	4217	(2.1)	4407	(2.1)	2997	(1.6)	3242	(1.6)
Heart disease, No. (%)	6886	(3.7)	8190	(4.3)	8322	(4.2)	8441	(4.1)	8810	(4.3)	5644	(3.0)	6102	(3.0)
Renal disease, No. (%)	1175	(0.6)	325	(0.2)	387	(0.2)	812	(0.4)	1130	(0.5)	766	(0.4)	896	(0.4)
Hypertension therapy, No. (%)	58,773	(35.5)	63,700	(33.7)	65,917	(32.9)	67,632	(33.1)	67,761	(33.0)	47,570	(29.0)	53,001	(29.4)
Diabetes therapy, No. (%)	13,613	(8.2)	13,730	(7.3)	14,965	(7.5)	15,781	(7.7)	15,883	(7.7)	12,456	(7.6)	13,845	(7.7)
Hyperlipidemia therapy, No. (%)	41,693	(25.2)	46,290	(24.5)	48,233	(24.1)	51,014	(25.0)	52,517	(25.6)	39,124	(23.8)	44,176	(24.5)
Current heart failure, No. (%)	5068	(2.6)	4944	(2.6)	5074	(2.5)	5108	(2.5)	5221	(2.5)	4766	(2.6)	5391	(2.7)

SD, standard deviation.

^a^This includes 3.9% of residents’ registries outside Fukushima Prefecture.

### Prevalence and hospitalization among age groups

**Fig. 1:**
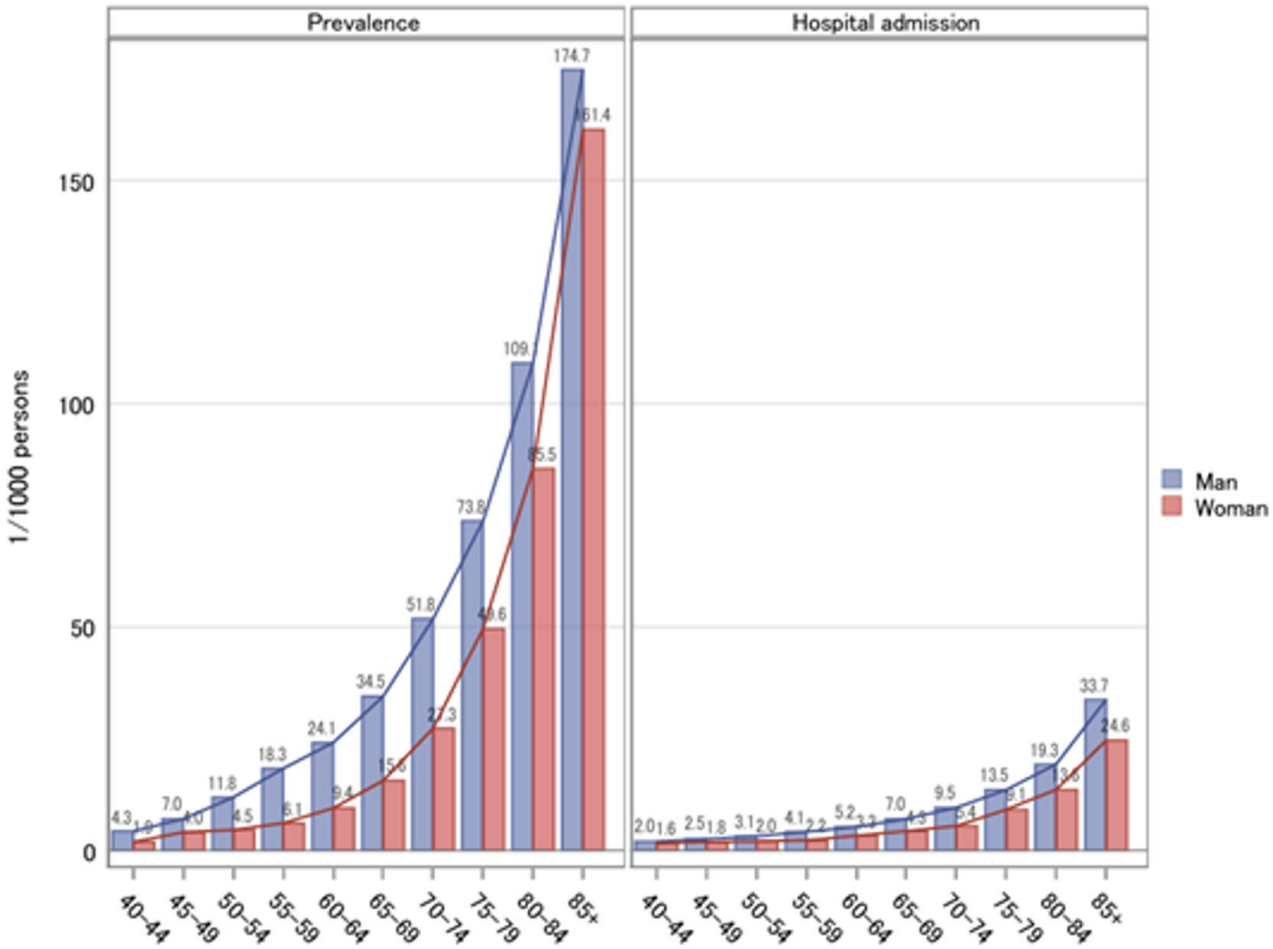
Mean prevalence and hospitalization rates of heart failure among annual health checkup participants aged 40–85 + years in Fukushima, 2015–2021.

Among participants aged ≥ 40 years, the overall prevalence per 1000 persons was 37.0 in men and 25.9 in women; the overall hospitalization per 1000 persons was 7.4 in men and 5.3 in women. As age increased, the prevalence and hospitalization rates increased in both sexes and were higher in men than in women in all age groups (Fig. 1). The sex disparity in the prevalence rate was highest at ages 50–64 years, and men were twice as likely as women. In addition, the mean number of hospitalizations per patient with HF was 1.95 and 1.42 times in men aged 40–59 and 60–85 + years, respectively, and 4.16 and 1.83 times in women in the corresponding age groups.

### Trends of age-standardized prevalence rates

**Fig. 2:**
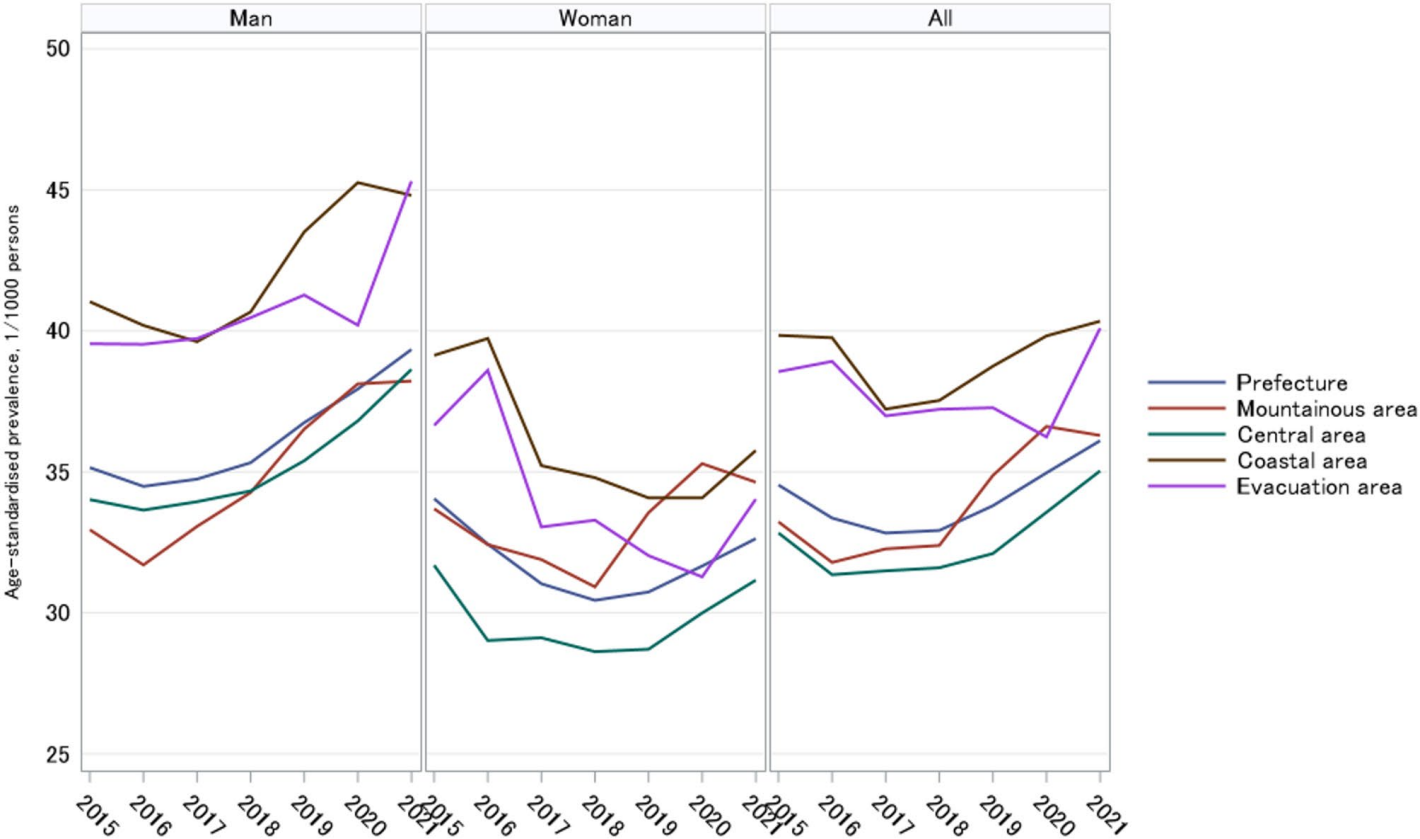
Age-standardized prevalence rates of heart failure in Fukushima and its subareas, 2015–2021. Compared with the whole prefecture, the prevalence was higher among men and among residents of the coastal and evacuation areas, whereas it was lower among women and those in the central area. Across periods, relative to 2015, prevalence was significantly lower in 2017 and 2018, but higher in 2021.

Figure 2 shows the age-standardized prevalence rates of HF over the years in the whole prefecture, mountainous, central, coastal, and evacuation areas. Overall, relative to the prefecture-wide mean, the standardized prevalence of HF was higher among men and among residents of the coastal and evacuation areas, whereas it was lower among women and those in the central area. Across periods, compared with 2015, prevalence was significantly lower in 2017 and 2018, but higher in 2021 (Table S1).

Table 2 summarizes the temporal trends and change points of HF prevalence rates in the different areas using Joinpoint regression analysis. Significantly increased annual HF prevalence rates were observed in men across all subareas in 2015 and 2021, with the largest increase in the mountainous area (1.15%) and the smallest in the evacuation area (0.72%). These upward trends were consistent with increases in the second segmented period: from 2017 in the coastal (APC = 1.51%) and mountainous areas (APC = 1.52%); from 2018 in the prefecture overall (APC = 1.20%) and the central area (APC = 1.47%); and from 2019 in the evacuation area (APC = 1.92%). In contrast, among prefecture women, a declining trend was observed before 2018 (APC = − 1.25%, *P* = 0.001), which shifted to an increasing trend after 2018 (APC = 0.82%, *P* = 0.002). For the overall population, a significant increase was seen in the second period in the prefecture (APC = 1.16%, *P* = 0.011) and in the coastal area (APC = 0.77%, *P* = 0.020) after 2018. The only significant increasing trend was observed in the mountainous area (AAPC = 0.78%, *P* = 0.021).

**Table 2:** Observed age-standardized prevalence rates of heart failure (1/1000 persons) (age 40–85 + years) and changes over the years (2015–2021).

Area	Year	Men			Women			All		
		Rate^a^	APC	AAPC	Rate^#^	APC	AAPC	Rate^a^	APC	AAPC
Prefecture	2015	35.15	− 0.32	0.77^b^	34.05	− 1.25^b^	− 0.19	34.53	− 0.52	0.33
	2016	34.48			32.44			33.36		
	2017	34.74			31.03			32.83		
	2018	35.32	1.20^b^		30.44	0.82^b^		32.92	1.16^b^	
	2019	36.75			30.74			33.80		
	2020	37.94			31.66			34.96		
	2021	39.34			32.63			36.10		
Coastal area	2015	41.03	− 0.63	0.90^b^	39.13	− 1.52	− 0.78	39.84	− 1.51	0.12
	2016	40.19			39.73			39.76		
	2017	39.62	1.51		35.22			37.22		
	2018	40.67			34.79	1.05		37.53	0.77^b^	
	2019	43.51			34.08			38.75		
	2020	45.25			34.07			39.82		
	2021	44.81			35.76			40.34		
Central area	2015	34.02	0.08	0.78^b^	31.68	− 0.94	0.03	31.35	− 0.40	0.43
	2016	33.64			29.02			31.49		
	2017	33.94			29.12			31.60		
	2018	34.32	1.47^b^		28.62	0.96		32.10	1.24	
	2019	35.39			28.71			33.57		
	2020	36.81			29.99			35.03		
	2021	38.63			31.16			31.35		
Mountainous area	2015	32.95	0.23	1.15^b^	33.69	− 0.63	0.39	33.23	− 0.53	0.78^b^
	2016	31.70			32.42			31.79		
	2017	33.06	1.52^b^		31.89			32.27	1.28	
	2018	34.27			30.92	1.36		32.39		
	2019	36.52			33.55			34.87		
	2020	38.12			35.29			36.61		
	2021	38.22			34.63			36.29		
Evacuation	2015	39.54	0.24	0.72^b^	36.65	− 1.54	− 0.75	38.55	− 0.59	0.01
	2016	39.52			38.60			38.92		
	2017	39.73			33.04			36.99		
	2018	40.47			33.29			37.22		
	2019	41.27	1.92^b^		32.03	1.01		37.27	1.41	
	2020	40.20			31.28			36.24		
	2021	45.31			34.03			40.09		

^a^Prevalence rates were standardized using the 2015 Japan national census for persons aged ≥ 40 years. APC, annual percentage change; AAPC, average annual percentage change; HF, heart failure. ^b^Indicates that the APC differs significantly from zero at an alpha level of 0.05.

Figure S1 shows the age-standardized HF prevalence rates by insurance type among the health checkup participants. For the NHI participants, men had a significant increase, with an APC of 0.70% (*P* = 0.004) between 2012 and 2019 and an AAPC of 0.89% (*P* < 0.001) between 2012 and 2021. There was no significant change point in the APC and AAPC (0.45%, *P* < 0.001) between 2012 and 2021, and no significant change in the HF prevalence rate among women. Women with EHI had a declining prevalence trend, with an AAPC of -0.65% (*P* = 0.018) between 2015 and 2021. For LEHI participants, women had a significant declining trend during the 2012 and 2018 periods, with an APC of − 3.68% (*P* = 0.042), while the men and overall LEHI participants had no significant change in prevalence rates during the 2012 and 2021.

### Age-period-cohort analysis

Before performing the age-period-cohort analysis, graphs of crude prevalence rates of the two components were plotted for the age-period (Fig. S2), age-cohort (Fig. S3), and period-cohort (Fig. S4).

**Fig. 3:**
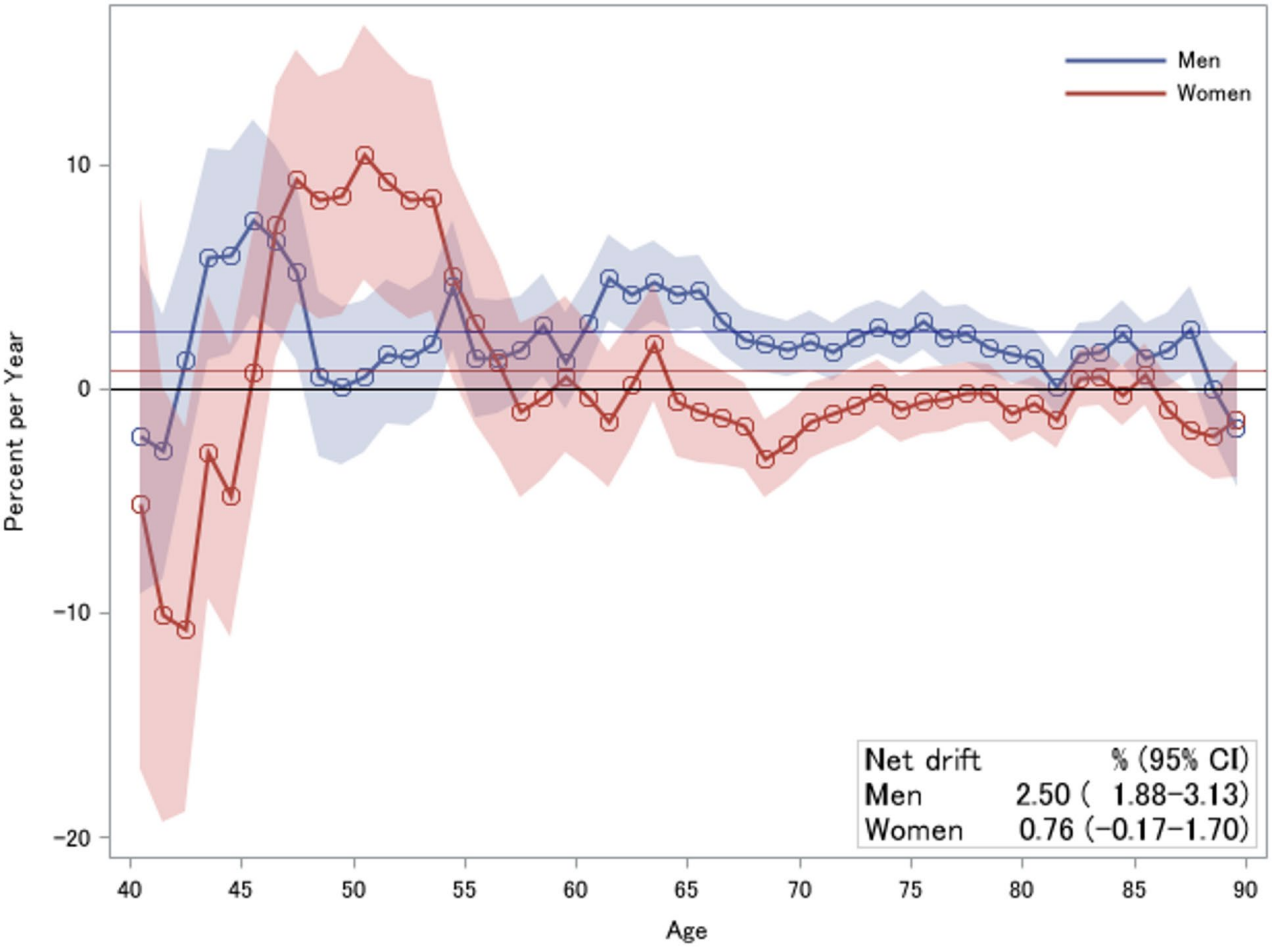
Local and net drift values of heart failure prevalence from age-period-cohort analysis, 2015–2021. Horizontal thin lines represent net drift values of 2.50% (95% confidence interval: 1.88–3.13%) for men and 0.76% (95% confidence interval: − 0.17–1.70%) for women.

Table S2 provides the parameters calculated in the age-period-cohort analysis. The net drift (overall APC) was significant in men (2.50%, 95% CI 1.88–3.13%) but not in women (0.76%, 95% CI − 0.17–1.70%), indicating that the prevalence rate increased in men. Local drift (APC in each age group) reflected age-specific variations, where values above 0 (*P* < 0.05) were observed in men. The highest values were observed in men aged 43–47 years and in women aged 46–53 years, indicating a rapid increase in the prevalence of HF (Fig. 3). For both sexes in the reference birth cohort, adjusted for the period effect, the expected HF prevalence per 1000 persons accelerated with age (Fig. S5), more noticeably in men than in women, as illustrated in the two-component graphs (Figs. S2 and S3).

**Fig. 4:**
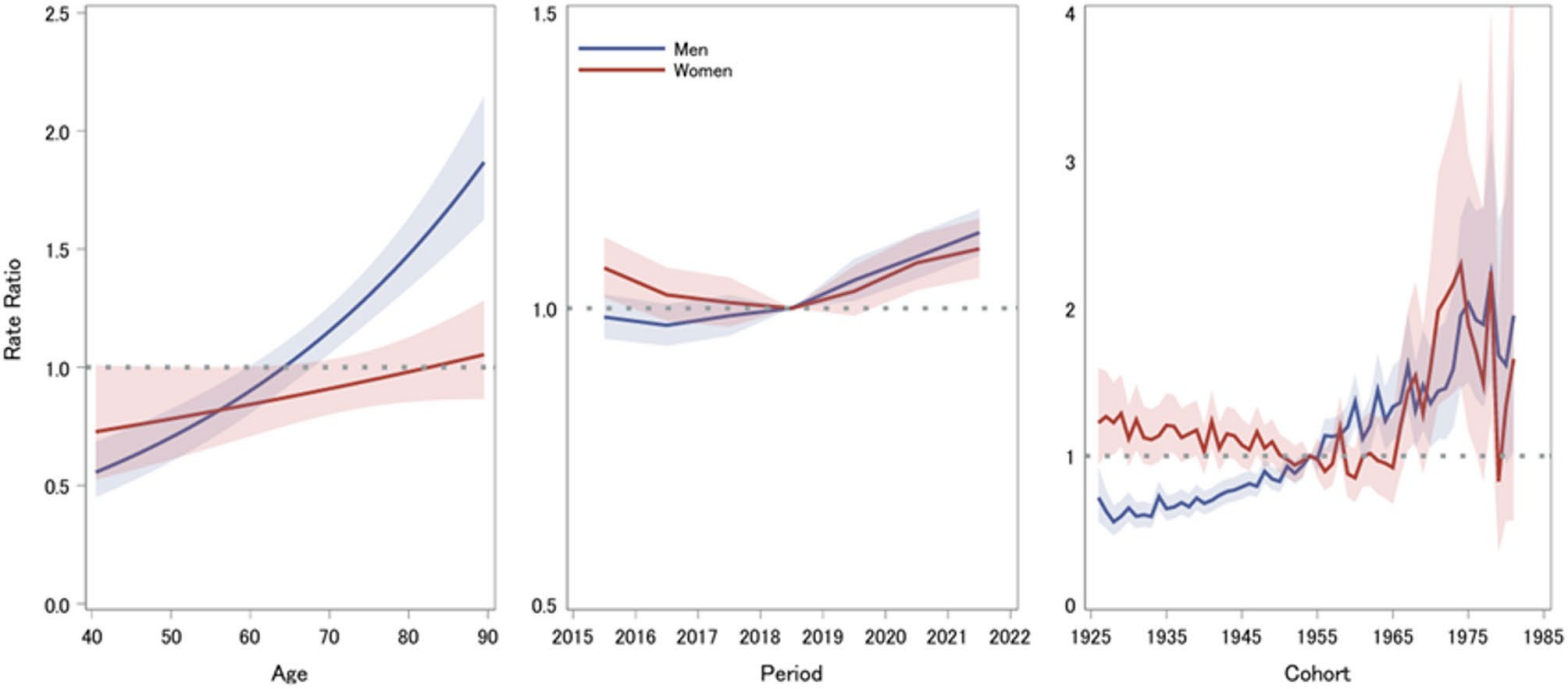
Rate ratios of heart failure prevalence for age groups (longitudinal vs. cross-sectional), period (adjusted for cohort), and cohort (adjusted for period) according to age-period-cohort (2015–2021).

Figure 4 shows the ratios of age-specific HF prevalence in periods relative to 2018 and the 1955 birth cohort. The prevalence significantly increased in men after 2018 and in women in 2015 and after 2018. The patterns along the calendar years are the same as those of the Joinpoint regression models. The men in the 1925–1975 birth cohorts showed a continuously increased risk (RRs were significantly lower before and higher after 1955, *P* < 0.05), while the women in the 1925–1960 birth cohorts showed a decreased risk and those in the 1960–1978 birth cohorts showed an inversely increased risk. RRs were significantly higher in the old (1927–1929, 1931, 1935, 1936, 1941, and 1958) and young cohorts (1967, 1968, 1970–1976, and 1978) compared to the 1955 cohort (*P* < 0.05).

In addition, in both sexes, the period deviations (departures from the overall linearity) were significantly higher in 2015 and 2021 and lower in 2018, indicating a difference from the expected rates.

## Discussion

This study provides new evidence on the prevalence of HF in Japan using real-world data. This prevalence was higher among men in the prefecture, with disparities in subareas, including the evacuation area.

Approximately 80% of the hospitals in Fukushima joined the Diagnostic Procedure Combination (DPC) system. In the participants aged below 75 years, the prevalence of HF was approximately double in men than in women, and the gap between sexes gradually closed with age. As reported in the Japanese Registry of All Cardiac and Vascular DPC database (JROAD-DPC), the peak age groups for HF are 60–69 years in men and 80–89 years in women^10^. The mean age of HF onset was 78.0 ± 12.5 years, with those aged > 75 years accounting for 68.9%^25^. Compared to the JROAD-DPC with 107,104 cases aged ≥ 40 years registered in 610 hospitals^2^, the proportion of HF cases in men in the FDB was lower in the 40–69 years age group but higher in the 70–89 years group. The proportions were lower in all age groups for women.

The CHART-2 Study reported that the mean age of patients with HF was 68.2 ± 12.3 years, and the prevalence of HF was 3.1%, 29.0%, 33.7%, and 34.2% for the < 40, 40–64, 65–74, and ≥ 75 years groups, respectively^13^. Our study had similar results for groups of those < 75 years old and higher prevalences for the ≥ 75 years groups. The disparity in hospital admission frequency among age groups and the relatively higher rate in women may be due to the impact of higher all-cause mortality in men than in women.

The prevalence rates from the annual health checkup study of participants aged ≥ 40 years were higher than those in the Global Burden of Disease study. Specifically, the age-standardized prevalence rate of 0.53% in Japan in 2019 was lower than that in China but higher than that in Singapore and Korea. This may be due to the inclusion of many young people and the reference population for standardization in the Global Burden of Disease study^8^. The prevalence rate in Fukushima was relatively similar to that in the Japan Medical Centre study (2.18–3.7% in those aged ≤ 74 years) and slightly lower than that in the Medical Data Vision study (6.5% in all ages)^9^. Internationally, due to the varying ages of study participants, our study results were probably higher than the prevalence rate of 1.4% in Taiwanese adults (≥ 20 years) in 2016 (increased by 0.63% in 2001)^26^ and 12.4/1000 persons in Korean adults (≥ 19 years) in 2014^27^. Detailed comparisons among age groups should be performed in future studies. The number of HF deaths (with reduced ejection fraction) may have declined, resulting in a high prevalence, even with stable incidence rates over the years^14,26^. Nevertheless, the prevalence rate in our study was similar to that in a meta-analysis of all HF types in developed countries (4.2%), which was considered a more realistic estimate in the general population^1,28^.

The decrease in participants in 2020 compared with other fiscal years was likely influenced by the COVID-19 pandemic, which restricted access to public health checkups. In addition, a recent study reported that patients hospitalized with HF during the pandemic experienced higher rates of in-hospital adverse events, including increased in-hospital mortality^29^. Therefore, the fluctuations in HF prevalence observed around 2020 in evacuation zones and mountainous areas may partly reflect reduced access to health checkups and increased HF morbidity and mortality during the pandemic. Further research is warranted to clarify these pandemic-related effects.

Sex-based differences in the risk, symptoms, and management of HF exist. The incidence of HF with preserved ejection fraction is higher in women than in men in Western countries. This is attributed to the cardiovascular effects of estrogen in women and varying risk factors between men and women^30^. Another study reported a similar incidence risk between men and women after adjusting for age and other risk factors^5^. In our study, the higher prevalence of lifestyle-related risks (smoking, alcohol consumption, physical inactivity, etc.) in men than in women may explain the higher prevalence of HF in men. The increase in HF prevalence rates in men, particularly in recent years, should be monitored intensively, and practical health promotion activities should be carried out urgently. Similarly, evidence from the CHART-1 and -2 studies suggests that the prevalence of HF will increase rapidly; thus, an effective strategy for HF prevention and patient management is necessary^3^.

The higher prevalence of HF in coastal areas and the rapidly increasing prevalence in mountainous areas may be attributed to lifestyle disparities^17^. For example, among men, the prevalence of overweight, hypertension, and dyslipidemia – risk factors for HF^5,31^ – was higher in the mountainous area than in the other two areas^16^. After 2015, the prevalence of metabolic syndrome in the mountainous area also became higher than that in the coastal area among women and residents aged 40–59 year^16^. These differences may reflect the epidemiological transitions across regions, communities, or ethnic groups within the same county, driven by ageing populations^32^, lifestyle changes, and the impact of disasters in Fukushima. Although we did not observe a significant difference in HF prevalence between NHI and EHI participants, the distribution of insurance enrolment changed over time: the proportion enrolled in NHI increased from 12.0% in 2015 to 16.1% in 2021, whereas the proportion enrolled in EHI decreased from 15.3 to 13.7% over the same period. Further studies must consider the impact of location, lifestyle, occupation, and socioeconomic factors on the prevalence of HF.

A continuous increase in the HF prevalence rate was observed in the temporal evacuation area, primarily located in the coastal area. Nakamura et al., in a population-based comprehensive registration study for hospitals, found that the incidence ratios for new-onset acute decompensated HF significantly increased in high-impact regions in 2011, 2013, and 2014, and the tsunami prolonged the increase^33^; this is supported by the higher HF prevalence in male post-disaster residents from 2015 to 2021 in our study. Cardiovascular factors, such as metabolic syndrome, showed a slowly increasing incidence in evacuation areas compared to non-evacuation areas, but recovery was not observed^19^. Moreover, except for ageing, psychiatric and mental health issues, such as post-traumatic stress, have an adverse prognostic impact in cardiovascular patients^34^.

Although Joinpoint regression can identify significant temporal trends in age-standardized rates and estimate the years of transition, it does not distinguish between influences that occur in specific periods for all age groups (i.e., period effects) versus effects associated with the year of birth (i.e., generational or birth cohort effects). The effect of age highlights the various risks associated with age at different stages of life. In this study, the estimated accelerated increase in HF prevalence demonstrated trends in an ageing society in Japan^14^, which might have a higher impact there than in other countries.

The period effect shows the variety of changes over time that affect the entire population, and the cohort effect represents risk factors and environmental exposures across the birth cohort. Based on one-year interval cohorts, we observed a decline in HF prevalence in women born between 1925 and 1960, but an increase in that of those born between 1960 and 1975. Meanwhile, we observed an increase in the prevalence of HF in almost all male cohorts. These lifespan sex disparities might be explained by the different lifestyle factors between men and women, including the increase in metabolic syndrome (i.e., overweight, hyperlipidemia, and hypertension) prevalence in men, and a significant decline in hypertension in women in Fukushima^17^. Notably, the Global Disease Burden study observed that the prevalence of HF attributable to hypertensive heart disease increased between 1990 and 2019^35^. These results suggest that control of risk factors for hypertension should be performed when prefectural authorities implement health promotion measures^15^. In addition, a decline in the mean age at onset of HF and an increasing trend of incident HF among young individuals (age ≤ 50 years) has been reported^1,36^. In our analysis, the inverse cohort effect in women since 1960 supports the findings of these reports.

One of the limitations of this study is the lack of data from the FDB before 2012, which prevented us from comparing our findings with those from before the triple disasters in March 2011. However, an NDB DPC health insurance claims study reported a continuously increasing number of HF cases from January 2011 to October 2015^9^; similar trends were observed in Fukushima, with a higher prevalence in men than in women in the early years. Second, because the claims data do not include the clinical stages (acute or chronic) and accurate mortality information on hospital discharge, we could not describe the HF burden in Fukushima. Meanwhile, the annual prevalence may have been higher if the death numbers for each year were not removed. Mortality due to HF and the sensitivity of diagnosis accuracy^9^ need to be addressed in further studies. Third, because health-checkup participation is approximately 56%, our estimates represent the population who undergo annual examinations, limiting generalizability to all residents. Previous studies have shown that individuals who do not attend health checkups tend to be older, more often male, of lower socioeconomic status, and to have less favorable lifestyle profiles than participants^37,38^. The primary FDB participants were those covered under NHI and EHI, while individuals with higher socioeconomic status and generally better health (e.g., employees of large corporations and public-sector workers covered by corporate health insurance societies or mutual aid associations) were not included. This may have contributed to an overestimation of HF prevalence. Conversely, individuals who are less health-conscious or have poorer health may be less likely to undergo health checkups, leading to underestimation. However, participation rates and participant characteristics have been stable over time, and health-exam datasets are widely used for chronic-disease prevalence research in Japan^39^. Therefore, both the level and temporal trends of HF prevalence in the examined population remain internally valid^40^. Furthermore, the inability to track changes in residence or insurance type over time (e.g., due to job changes or municipal relocation) may have introduced misclassification, potentially biasing the estimates in either direction. Fourth, a period of seven calendar years (2015–2021) may not have been sufficient to observe any additional possible change points in the temporal trends. Although period deviations were statistically significant, no distinct calendar-time event explained these changes. Given the short observation window, the deviations likely reflect modest year-to-year fluctuations rather than systematic shifts; thus, the period effects should be interpreted with caution. Additionally, although the cohort effect on increasing HF prevalence has been elucidated, the few young cohorts (e.g., 1978–1981) show uncertainty in both sexes.

## Conclusions

To conclude, our analysis revealed that HF prevalence rates varied in Fukushima between 2015 and 2021, with higher rates in men in the coastal area and the evacuation zone established after the disaster in March 2011. In contrast, rates increased continuously in men, particularly in the mountainous area. As pathological changes and modifiable risk factors for HF accrue over time, it is essential to maintain continuous monitoring and to conduct further research on the HF burden in Fukushima, particularly among individuals younger than 60 years, to inform timely prevention and targeted intervention strategies.

## Supplementary Information

Below is the link to the electronic supplementary material.

Supplementary Material 1

## Data Availability

The datasets generated and/or analyzed during the current study are not publicly available because of confidentiality, but are available from the corresponding author upon reasonable request and with permission of the Fukushima Government.
