# Supplementary material for: Trends in heart failure prevalence in post-disaster Fukushima residents 2015–2021
Source: Sci Rep. 2026 Jan 14;16:5222. doi: 10.1038/s41598-026-36032-0 (PMC12881415; doi:10.1038/s41598-026-36032-0)
Supplement: Supplementary file 1 — Supplementary Material 1 [file 41598_2026_36032_MOESM1_ESM.docx]

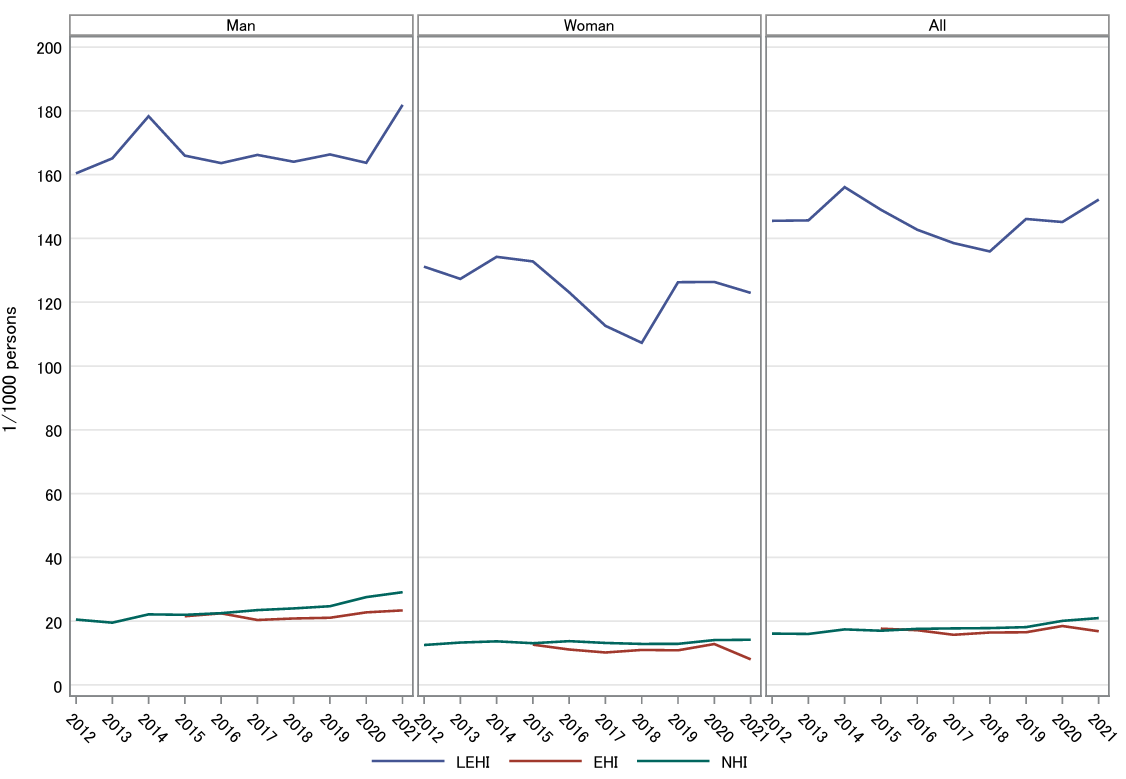


**Figure S1.** Age-standardized prevalence rates of heart failure among beneficiaries of national health insurance (40–75 years, 2012–2021), employee health insurance (40–75 years, 2015–2021), and late elderly health insurance (65–85+ years, 2012–2021).


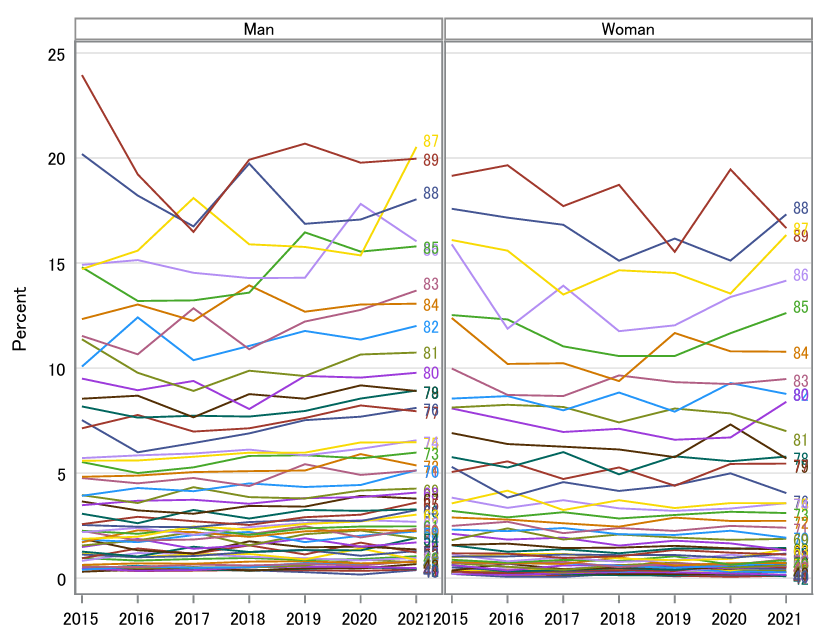


**Figure S2.** Prevalence rates of heart failure by age group across calendar years, 2015–2021.


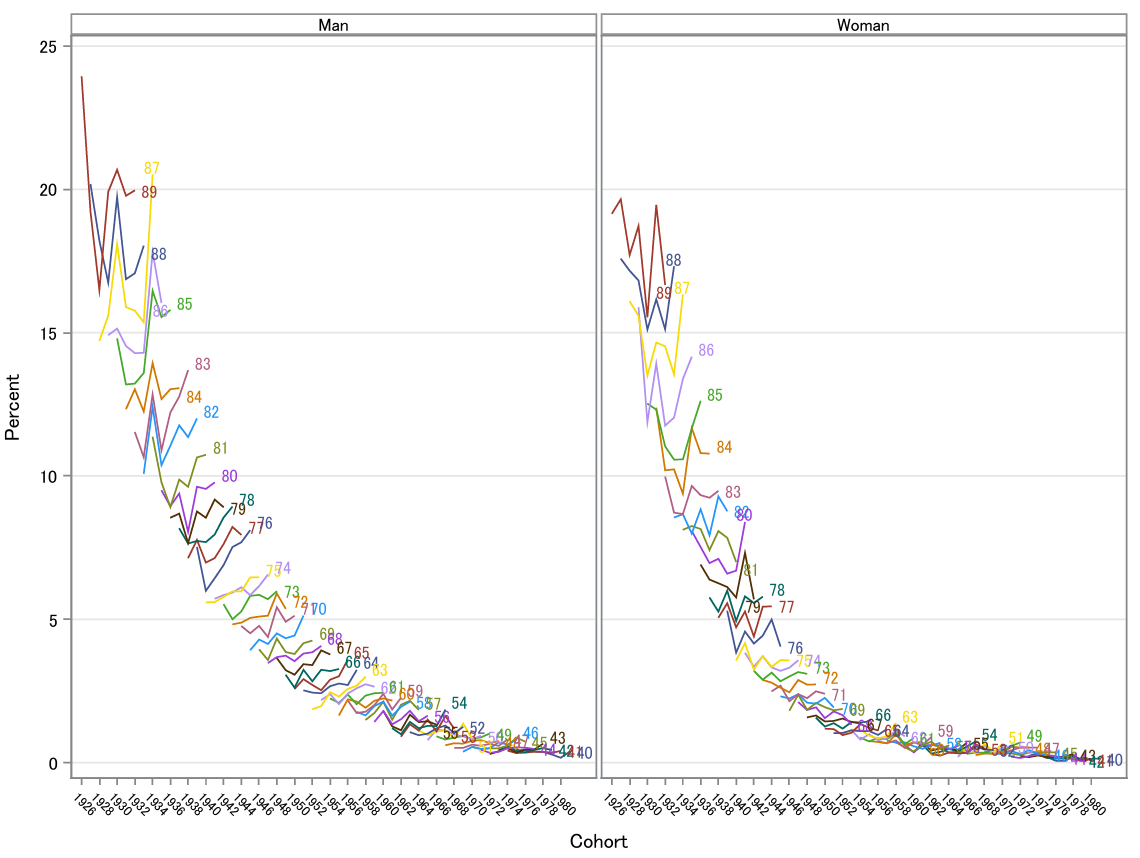


**Figure S3.** Prevalence rates of heart failure by age group across birth cohorts, 1926–1981.


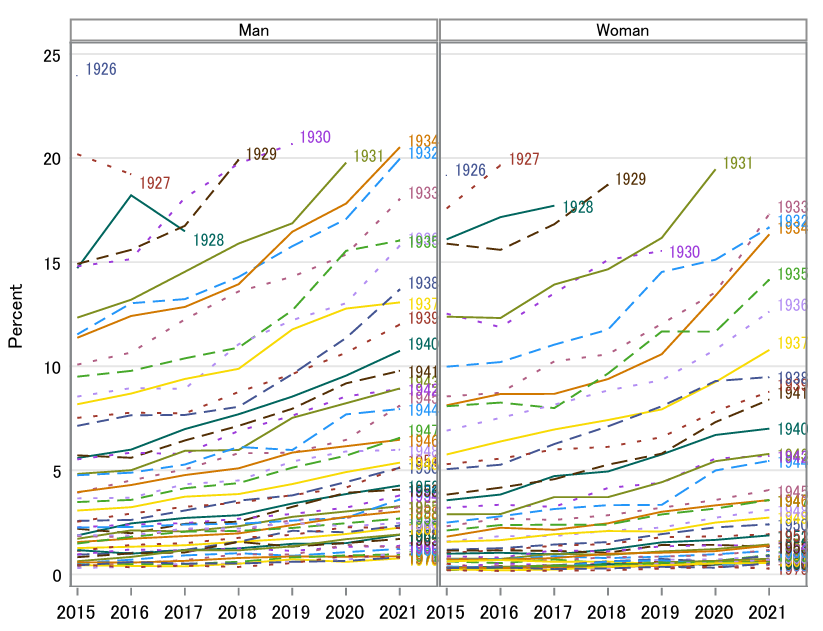


**Figure S4.** Prevalence rates of heart failure by birth cohort across calendar years, 2015–2021.

| Variables | β (95% Confidence Interval) | *P*-value |
| --- | --- | --- |
| All | Ref. |  |
| Men | 2.40 (1.68, 3.12) | <0.001 |
| Women | -2.49 (-3.20, -1.77) | <0.001 |
|  |  |  |
| 2015 | Ref. |  |
| 2016 | -0.66 (-1.76, 0.43) | 0.235 |
| 2017 | -1.65 (-2.74, -0.55) | 0.003 |
| 2018 | -1.47 (-2.57, -0.38) | 0.008 |
| 2019 | -0.50 (-1.60, 0.59) | 0.368 |
| 2020 | 0.33 (-0.77, 1.42) | 0.556 |
| 2021 | 1.70 (0.60, 2.80) | 0.002 |
|  |  |  |
| Prefecture | Ref. |  |
| Central area | -1.53 (-2.46, -0.61) | 0.001 |
| Coastal area | 5.04 (4.12, 5.97) | <0.001 |
| Evacuation area | 3.57 (2.65, 4.50) | <0.001 |
| Mountainous area | -0.03 (-0.95, 0.90) | 0.956 |

**Table S1.** Overall difference of standardized heart failure prevalence rates among sex, subareas, and years.

| Null Hypothesis | Men | | |  | Women | | |
| --- | --- | --- | --- | --- | --- | --- | --- |
|  | Chi-square | *df* | *P*-value |  | Chi-square | *df* | *P*-value |
| Net Drift = 0 | 63.64 | 1 | <0.001 |  | 2.55 | 1 | 0.111 |
| All Age Deviations = 0 | 42.46 | 48 | 0.698 |  | 35.77 | 48 | 0.904 |
| All Period Deviations = 0 | 15.49 | 5 | 0.008 |  | 28.87 | 5 | <0.001 |
| All Cohort Deviations = 0 | 128.01 | 54 | <0.001 |  | 144.03 | 54 | <0.001 |
| All Period RR = 1 | 85.27 | 6 | <0.001 |  | 32.42 | 6 | <0.001 |
| All Cohort RR = 1 | 209.02 | 55 | <0.001 |  | 147.40 | 55 | <0.001 |
| All Local Drifts = Net Drift | 105.50 | 50 | <0.001 |  | 122.22 | 50 | <0.001 |

**Table S2.** Wald chi-squared tests for parameters in the age-period-cohort model, 2015–2021. *RR = rate ratio.


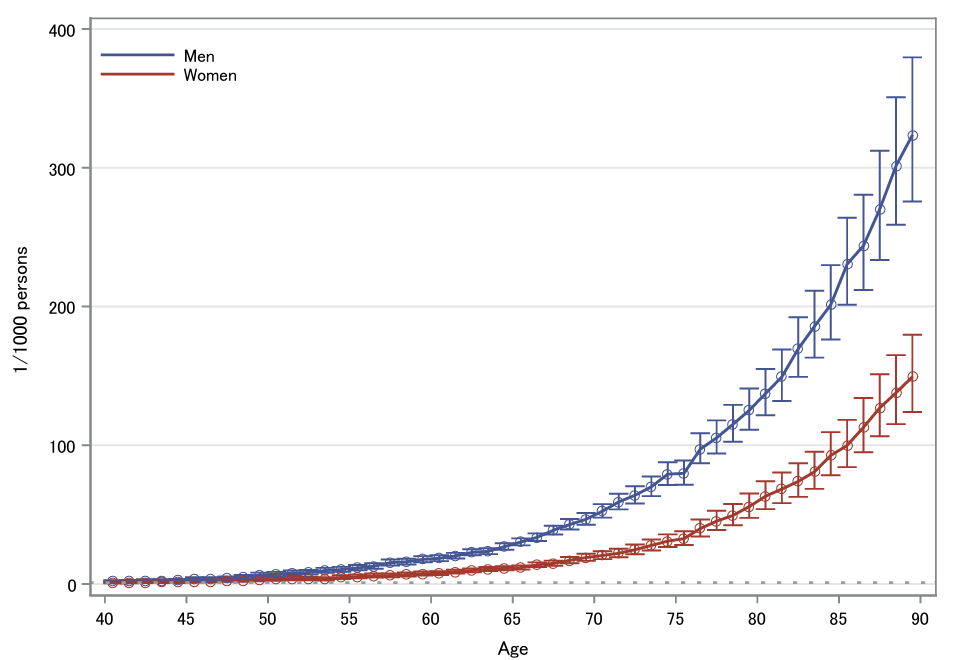


**Figure S5.** Estimated longitudinal curves of heart failure prevalence rates (adjusted for period effect in the reference cohort) from the age-period-cohort model, 2015–2021.
